# Pharmacogenetic variation influences sensory neuropathy occurrence in Southern Africans treated with stavudine-containing antiretroviral therapy

**DOI:** 10.1371/journal.pone.0204111

**Published:** 2018-10-01

**Authors:** Marvin Blessings Moketla, Antonia L. Wadley, Peter Kamerman, Debra de Assis Rosa

**Affiliations:** 1 School of Molecular and Cell Biology, Faculty of Science, University of the Witwatersrand, Johannesburg, South Africa; 2 Brain Function Research Group, School of Physiology, Faculty of Health Science, University of the Witwatersrand, Johannesburg, South Africa; 3 School of Pharmacy and Biomedical Sciences, Faculty of Health Sciences, Curtin University, Perth, Australia; University of Bristol, UNITED KINGDOM

## Abstract

**Background:**

The use of the HIV antiretroviral drug stavudine (d4T), a thymidine analogue, is associated with the development of mitochondrial toxicities such as sensory neuropathy (SN). Genetic variation in genes relating to d4T transport and metabolism, as well as genetic variation in the thymidine synthesis pathway, could influence occurrence of d4T-related toxicity.

**Methods:**

We examined this hypothesis in a cohort of HIV-positive South African adults exposed to d4T, including 143 cases with SN and 120 controls without SN. Ten SNPs in four genes associated with stavudine transport, and 16 SNPs in seven genes of the thymidine synthesis / phosphorylation pathway were genotyped using Agena mass spectrometry methods. Associations between sensory neuropathy and genetic variants were evaluated using PLINK by univariate and multivariable analyses.

**Results:**

Age and height were significantly associated with SN occurrence. Using logistic regression with age and height as covariates, and uncorrected empirical p-values, genetic variation in *SLC28A1*, *SAMHD1*, *MTHFR* and *RRM2B* was associated with SN in South Africans using d4T.

**Conclusion:**

Variation in genes relating to d4T transport and metabolism, as well as genetic variation in the thymidine synthesis pathway may influence occurrence of d4T-related SN. These data contribute to the characterisation of African pharmacogenetic variation and its role in adverse response to antiretroviral therapy.

## Introduction

The use of antiretroviral therapy (ART) has changed the natural history of human immunodeficiency virus (HIV) infection into a manageable chronic disease requiring long-term treatment. ART stops or delays progression to AIDS by suppressing HIV viraemia to undetectable levels, thereby improving CD4+ T cell counts, and restoring immune function [1]. However despite the benefits of ART, a substantial percentage of treated individuals experience adverse drug reactions such as off-target toxicities and hypersensitivity reactions. For example, in South Africa, the thymidine analogue stavudine (d4T) that was used in a first line ART regimen from 2004 to 2010 [2, 3], was associated with high incidence of mitochondrial toxicities, including sensory neuropathy (SN), lactic acidosis, and lipodystrophy [4].

Sensory neuropathy, the most frequent of the d4T adverse effects, is typically experienced as bilateral pain, pins-and-needles, and/or loss of sensation in a “stocking and glove” distribution on the body [5]. In two South African cohorts, more than half of individuals on d4T-containing regimens experienced SN [6, 7]. Drug-induced side effects often cause drug regimen switches. South Africans substituted d4T for other NRTIs in 21% of patients by three years of treatment [8] and in another study, at a high rate of 17.9 changes per 100 person-years [9] due to adverse d4T effects. Due to d4T’s toxicity profile, South African HIV treatment guidelines changed in 2010, in line with international recommendations, replacing d4T in the first line regimen with tenofovir [3].

Factors repeatedly associated with the development of SN after d4T use includes increased age and increased height [5]. But because not all individuals experience adverse drug reactions when treated with HIV antiretrovirals, there also may be a genetic predisposition to occurrence of side-effects. To date, investigations into genetic risk factors for SN have focused on genes related to mitochondrial function [10, 11] and inflammation [12, 13, 14]. However variation in the enzymes responsible for d4T transport and drug metabolism, which may influence susceptibility to d4T adverse reactions, has not yet been investigated. These “ADME” genes (affecting drug absorption, distribution, metabolism and elimination) [15] are often the focus of pharmacogenetic studies which aim to identify host genetic factors influencing drug efficacy and toxicity [16, 17]. Variation in ADME genes could influence d4T toxicity.

Stavudine is transported into cells by the OAT1, CNT1, CNT3 and ENT3 membrane transporters [18–22], encoded by *SLC22A1*, *SLC28A1*, *SLC28A3* and *SLC29A3* genes respectively. Efflux is controlled by MDR1, BCRP and MRP5 transporters [18, 23] encoded by the *ABCB1*, *ABCG2* and *ABCG5* genes respectively. Intracellularly, stavudine-5’- triphosphate (d4T-TP), the active form of stavudine, is produced by sequential phosphorylation steps by the action of enzymes thymidine kinase, thymidylate kinase, and nucleoside diphosphate kinase [24], encoded by genes *TK1*, *TK2*, *DTYMK* and *NME1*. Enzymes of the UDP-glucuronosyltransferase (UGT) family perform phase II metabolism of d4T [24]. Variation in any of these ADME genes could potentially relate to d4T efficacy and toxicity.

The efficacy and toxicity of d4T (or d4T-TP) can also be influenced by the naturally occurring levels of thymidine triphosphate, with which d4T completes for incorporation into the nascent HIV DNA strand. Decreased endogenous deoxyribonucleotide pools result in higher incorporation of the NRTI phosphate [25] and could cause both increased NRTI efficacy and increased NRTI toxicity. Selvaraj et al. [26] found that HIV-positive individuals on ART who had clinical phenotypes associated with NRTI-induced mitochondrial toxicity, had lower ribonucleotide (RN) and deoxyribonucleotide (dRN) pools compared to infected individuals on ART who did not have mitochondrial toxicity. The amount of thymidine triphosphate (dTTP) present in a cell is governed by the rate of *de novo* and salvage thymidine synthesis and phosphorylation, and therefore by variation in enzymes along these pathways. Key enzymes of the thymidine *de novo* synthesis pathway include ribonucleotide reductase (RNR), nucleoside diphosphate kinase, dUTPase, thymidylate synthetase encoded by genes *RRM1*, *RRM2*, *RRM2b*, *NME1*, *DUT* and *TYMS*. The thymidine salvage and phosphorylation pathways involve the same enzymes that phosphorylate d4T (genes *TK1*, *TK2*, *DTYMK* and *NME1*). dTTP, but not d4T, is dephosphorylated by enzyme SAMHD1 [27]. The production of dTMP by thymidylate synthetase is tightly linked to the folate / methionine cycle, and therefore enzymes dihydrofolate reductase (DHFR) and 5,10-methylene-THF-reductase (MTHFR) also play a critical role in regulating thymidine production. Variation in any of these genes regulating thymidine synthesis could also potentially affect efficacy and toxicity of d4T.

In summary, genetic variation in ADME genes relating to d4T transport and metabolism, as well as genetic variation in the thymidine synthesis pathways, could influence d4T-related toxicity. We examined this hypothesis in a black Southern African cohort exposed to d4T and phenotyped for sensory neuropathy, a common d4T-related toxicity.

## Materials and methods

### Study design and ethics statement

Blood samples were collected from adults attending the Virology Clinic of the Charlotte Maxeke Johannesburg Academic Hospital, South Africa, between 2008 and 2009, as described in [7, 12]. Sample collection and genetics study protocols were approved by the Human Research Ethics Committee of the University of Witwatersrand (M080220 and M150459) and all participants gave written informed consent. Demographic data were collected, including ethnicity, age, gender and height. Participants who self-identified as black African and had no unrelated cause of neuropathy (such as diabetes or vitamin B12 deficiency) were included in genetic analyses. All participants had been exposed to d4T, had been on ART for at least six months, but were not necessarily using d4T at the time of sampling.

SN in participants was assessed using the Brief Neuropathy Screening Tool [28] which is validated for the assessment of HIV-associated sensory neuropathy. This tool involved assessment of signs and symptoms of sensory neuropathy. Signs included absent ankle reflexes and reduced vibration sense in the great toe (using a 128Hz tuning fork, vibration sense of less than 10 seconds were regarded as reduced function), and symptoms included pain and paraesthesias (pins and needles, tingling and numbness). Sensory neuropathy was defined according to [28], namely the bilateral presence of at least one sign and one symptom.

### DNA extraction and genotyping

DNA was extracted from saliva in 39 samples using a QIAamp DNA mini kit (Qiagen) or from whole blood for the remainder of the samples using the salting out method [29]. Of the original 342 samples analysed by [12], 263 DNA samples were still available for use in the current study, including DNA from 143 cases with SN and 120 controls without SN. While 95% of the cohort were black South Africans, thirteen (5%) of the DNA samples were from individuals from neighbouring countries (Zimbabwe, Malawi or Mozambique). These samples were included in this study since black Africans speaking Bantu languages from all of these countries are considered to have similar genetic ancestry due to population migrations within the last 3 000 years [30].

A total of 26 single nucleotide polymorphisms (SNPs) in twelve candidate genes were examined in this study. The genes included two genes encoding proteins that facilitate d4T entry into cells (*SLC28A1*, *SLC28A3*), two genes encoding proteins that facilitate d4T efflux from cells (*ABCG2*, *ABCC5*), and eight genes from the thymidine synthesis / d4T phosphorylation pathways (*TK2*, *RRM1*, *RRM2*, *RRM2b*, *MTHFR*, *DHFR*, *SAMHD1*, *SLC19A1*). Genes, and SNPs in these genes, were selected based on descriptions of SNP functional effects (such as changes in gene expression) or relevant phenotypic associations (such as association with adverse drug reactions) in the literature. SNPs were further selected if they had minor allele frequency >5% in Kenyan Luhya samples from the 1000 Genomes database [31] and according to suitability for multiplex analysis on the genotyping platform. Genotyping was performed in two multiplexes using Agena MassArray mass spectrometry [32] at Inqaba Biotec (Pretoria, South Africa).

### Statistical analyses

SNPs or DNA samples with >10% missing data were excluded from analysis and Hardy-Weinberg equilibrium was assessed. Demographic characteristics in the cases vs. controls were compared by chi-squared or Mann-Whitney tests and significant differences were used as covariates in the multivariable analyses of genetic data. Allele, genotype and haplotype frequencies were assessed using gPLINK v.1.07 [33] and Haploview v.4.2 [34]. All possible 3-SNP haplotypes across each gene or chromosome under investigation were created using the sliding window option in gPLINK. Minor allele frequency (MAF) in Southern Africans was compared to MAF in Kenyan Luhya samples from the 1000 Genomes database using Fishers exact tests.

The associations between sensory neuropathy and genetic variants (alleles, genotypes or haplotypes) were evaluated using gPLINK using chi-squared or Fishers tests for univariate analysis and using logistic regression for multivariable analysis. Two different P values were reported arising from logistic regression analyses: firstly, P values based on the logistic regression coefficient t-statistic and secondly, empirical P values calculated by the max(T) permutation method with 10 000 simulations,. Empirical P values (Pemp) less than 0.05 were considered statistically significant. These Pemp values are considered more robust than the t -test p values since permutation methods do not make distributional assumptions but rather generate the reference distribution of the tests statistic from the data. The logistic regression coefficient t- statistic data were used to calculate odds ratios and 95% confidence intervals, since this cannot be done using permutation methods. SNPs with OR>1 were considered to be possible risk factors for SN occurrence, while SNPs with OR<1 were considered to be protective. No corrections for multiple testing were considered in this exploratory study. All associations were tested as the minor allele compared with wild-type.

## Results

### Age and height are associated with HIV-SN after d4T use

Characteristics of the study cohort are presented in Table 1. The comparison of demographic characteristics between cases and controls showed that increased age and increased height were significantly associated with SN occurrence in this cohort, as previously shown for the parent cohort [12].

**Table 1:** Demographic and clinical characteristics of the cohort.

	**whole cohort**	**Cases** **with SN**	**Controls** **without SN**	**P value**
**sample size**	263	143	120	-
**females, n (%)**	205 (78%)	110 (77%)	95 (79%)	0.333
**age, years (std dev)**	38	41 (7.7)	36 (7.2)	**<0.0001**
**height, cm (std dev)**	158.1	159.4 (8.7)	156.4 (7.4)	**0.007**
**weight, kg**	68.61	69.56	67.49	0.231
**body mass index (BMI)**	27.55	27.45	27.66	0.970
**months since HIV diagnosis**	52	54	49	0.538
**Median CD4+ T-cell count at sampling (on ART) cells/μl**	398 (27–1091)	406(27–1091)	393(45–1079)	0.407
**Median CD4+ T-cell count nadir (pre-ART) cells/μl (range)**	100 (1–403)	101 (1–253)	96 (2–403)	0.762
**months on d4T**	20	20	19	0.770

All values are means unless otherwise indicated.

n: sample size; std dev: standard deviation

### Characterisation of variation in thymidine synthesis and d4T ADME genes in black Southern Africans

Genotyping was successful for all 26 SNPs in 243 samples after quality control procedures (134 cases and 109 controls), and all loci were in Hardy Weinberg equilibrium (P>0.05). Minor allele frequencies (MAF) ranged from 0.021 (*RRM1* rs12806698) to 0.477 (*RRM1* rs1465952) as shown in Table 2. The MAF of seven SNPs in the Southern African cohort differed significantly (P<0.05) from MAF in Kenya Luhya, including three SNPs in the d4T drug transporter gene and four SNPs in genes of the thymidine synthesis pathway, *RRM2*, *RRM1* and *TK2* (Table 2). The MAF for rs1801131 and rs1801133 in *MTHFR* reported here were also not significantly different from the MAF reported in a previous South African black cohort with unknown HIV infection status [35]. For the remainder of the SNPs, allele frequencies have not been reported previously in South or Southern African black populations.

**Table 2:** Minor allele frequencies (MAF) in 243 black Southern Africans (SA) compared to MAF in 99 Luhya from Kenya (LWK).

Chromosome	Gene	SNP	Major allele	Minor allele	MAF in SA (n = 243, 2n = 486)	MAF in LWK (n = 99, 2n = 198)	P value
**3**	*ABCC5*	rs3749442	G	A	0.208	0.237	0.413
**4**	*ABCG2*	rs2725252	C	A	0.193	0.222	0.402
**4**	*ABCG2*	rs2622604	C	T	0.132	0.126	0.901
**4**	*ABCG2*	rs12505410	T	G	0.122	0.126	0.898
**4**	*ABCG2*	rs3114018	A	C	0.419	0.333	**0.045**
**9**	*SLC28A3*	rs4877847	A	C	0.470	0.475	0.932
**9**	*SLC28A3*	rs7853758	G	A	0.5	0.303	**<0.0001**
**15**	*SLC28A1*	rs2242046	G	A	0.025	0.061	**0.037**
**15**	*SLC28A1*	rs2290272	G	A	0.242	0.172	0.053
**15**	*SLC28A1*	rs8187758	C	A	0.155	0.131	0.477
**11**	*RRM1*	rs1465952	G	A	0.477	0.389	**0.042**
**11**	*RRM1*	rs11030918	T	C	0.20	0.167	0.335
**11**	*RRM1*	rs12806698	C	A	0.021	0.015	0.766
**11**	*RRM1*	rs1042927	A	C	0.181	0.263	**0.021**
**2**	*RRM2*	rs7574663	C	G	0.084	0.146	**0.018**
**8**	*RRM2B*	rs16918482	A	C	0.064	0.076	0.615
**16**	*TK2*	rs3743712	A	G	0.391	0.369	0.604
**16**	*TK2*	rs11859474	G	A	0.263	0.293	0.449
**16**	*TK2*	rs2288399	A	G	0.152	0.081	**0.012**
**1**	*MTHFR*	rs1801131	T	G	0.144	0.187	0.165
**1**	*MTHFR*	rs1801133	G	A	0.070	0.071	1.000
**5**	*DHFR*	rs1650723	C	T	0.062	0.071	0.731
**20**	*SAMHD1*	rs8124728	G	A	0.169	0.152	0.649
**20**	*SAMHD1*	rs1291142	A	G	0.231	0.237	0.921
**20**	*SAMHD1*	rs1891643	A	G	0.187	0.207	0.593
**21**	*SLC19A1*	rs1051266	T	C	0.311	0.308	1.000

All SNPs are on forward strand. P values in bold indicate P<0.05, Fisher’s exact test

Linkage disequilibrium (LD) was analysed in seven genes where more than one SNP was genotyped. Complete linkage (D’ = 1.000) was found among the following SNPs: rs1465952, rs11030918 and rs12806698 in *RRM1*; rs3743712 and rs11859474 in *TK2*; rs8124728, rs1291142 and rs1891643 in *SAMHD1*; rs12505410 and rs2725252 in *ABCG2*. In addition, D’ was 0.961 between rs2290272 and rs8187758 in *SLC28A1*. SNPs in *MTHFR* and in *SLC28A3* were not in significant LD with each other (D’ <0.3).

### Associations between ADME and thymidine synthesis genetic variants and sensory neuropathy

For all SNPs in univariate analyses, the minor allele frequency did not differ significantly between cases and controls. S*LC28A1* rs8187758 and *SAMHD1* r8124728 were significantly associated with SN in univariate genotypic models (Pemp = 0.011 and Pemp = 0.038, Table 3). In multivariable analyses taking age and height into account, rs8187758 in *SLC28A1* was no longer significantly associated with SN. Variation in *SAMHD1* remained associated with SN in multivariable analysis, including rs8124728 (Pemp = 0.013), rs1891643 (Pemp = 0.017) and haplotype GGA (Pemp = 0.015, Table 3). Two SNPs in the thymidine synthesis pathway, rs1801131 in *MTHFR* and rs16918482 in *RRM2B* were associated with SN in a multivariable genotypic and recessive models (Pemp = <0.002, Table 3).

**Table 3:** Gene polymorphisms significantly associated with SN after d4T use.

Gene	SNP	Univariate analysis Pemp value ^1^	Model of association	Multivariable analysis Pemp value^1^^,^^2^	Multivariable analysis P value^2^^,^ ^3^	OR in multivariable analysis (95% CI) ^4^	(Minor allele) direction of effect
*SLC28A1*	rs8187758	**0.011**	genotypic	0.249	0.060	1.89 (0.61–5.86)	(A) risk
*SAMHD1*	rs8124728	**0.038**	genotypic	**0.018**	0.709	0.45 (0.19–1.05)	(A) protective
*SAMHD1*	rs8124728	0.092	recessive	**0.014**	**0.047**	0.18 (0.03–0.98)	(A) protective
*SAMHD1*	rs1891643	0.280	recessive	**0.025**	0.075	5.08 (0.85–30.44)	(G) risk
*SAMHD1*	rs8124728, rs1291142, rs1891643	0.073	haplotype GGA	**0.015**	**0.048**	0.33 (0.07–0.98)	(GGA) protective
*MTHFR*	rs1801131	0.198	recessive	**0.001**	0.146	0.12 (0.01–2.07)	(G) protective
*MTHFR*	rs1801131	0.244	genotypic	**0.002**	0.224	0.33 (0.08–1.39)	(G) protective
*RRM2B*	rs16918482	0.509	recessive	**0.0003**	0.772	0.65 (0.04–11.60)	(C) protective
*RRM2B*	rs16918482	0.416	genotypic	**0.002**	0.954	0.81 (0.19–3.26)	(C) protective

1 Pemp values were from permutation analysis during multiple logistic regression using 10 000 simulations; Uncorrected Pemp values < 0.05 were considered significant and are shown in bold.

2 multivariable tests took into account age and height as covariates

3 P values were standard results from coefficient t-tests during multiple logistic regression and were used to calculate OR and direction of effect.

4 OR: odds ratio; CI: confidence interval

These Pemp values were not corrected for multiple testing and may therefore be false positives; we have used them as hypothesis- generating associations. The Pemp values calculated using permutation models were considered more robust than the p values used for calculation of odds ratios (Table 3). Considering the odds ratios generated during logistic regression, the recessive genotype of *SAMHD1* rs1891643 may be considered as a possible risk factor for SN occurrence (OR = 5.089) whereas all other genotypes described above as significant in multivariable analysis may be protective against SN (OR<1, Table 3). Weak effect sizes were noted for these loci. The complete results for multivariable analysis can be found in S1 Table.

## Discussion

Pharmacogenetics is concerned with characterising individual genetic variability and understanding how it affects response to, and side-effects arising from treatment. We explored variation in d4T ADME genes, and variation in genes of the thymidine synthesis pathway, and their association with SN in a cohort of HIV-positive South African adults treated with d4T-containing ART. We found that genetic variants of the *SLC28A1*, *SAMHD1*, *MTHFR* and *RRM2B* genes were associated with SN in this cohort using uncorrected Pemp values. This is the first report of associations between genetic variation in *SAMHD1 a*nd *RRM2B* and ART-induced toxicity. Our findings support previously reported associations between variation in *SLC28A1* and *MTHFR* and toxicity of ART.

*SLC28A1* encodes the CNT1 membrane transport protein which assists with d4T influx into the cell. A previous study found an association in Ethiopians between variation in *SLC28A1* and dose-toxicity of zidovudine (AZT), another thymidine analogue used in ART [36]. However the associated variant in that study was rs2242046, which was not associated with SN in the current study, and not in linkage with significant SNPs in the current study. In this study, *SLC28A1* SNP rs8187758 was associated in univariate analysis, but not multivariable analysis, with SN occurrence. This SNP has been previously reported to increase thymidine uptake [37], but its effect on d4T is not known. If only thymidine uptake and not d4T uptake is increased, this should potentially cause decreased d4T toxicity, however if this SNP also increases d4T influx then the protective effect would be ablated. SNP rs8187758 was in high LD with rs2290272 in our cohort, which has been shown to cause reduced expression of CNT1 and lowered affinity for substrates [38]; the effects of combinations of these SNPs on d4T uptake is not known.

The SAMHD1 enzyme dephosphorylates dTTP but does not hydrolyse active d4T back to its unphosphorylated form [27]. SAMHD1 activity may therefore increase efficacy or toxicity of thymidine analogues by reducing competition with intracellular dNTP [39]. We found significant associations between *SAMHD1* genotypes and haplotypes involving SNPs rs8124728 and rs1891643, and SN in our cohort in both univariate and multivariable analyses. These SNPs are located in an intron and the 3’ UTR of the gene respectively with unknown function. They were in complete LD with rs1291142, a SNP described as causing decreased SAMHD1 expression [40]. However rs1291142 itself was not found to be associated with SN in this study, and SNP rs1891643 was seen to cause a 5-fold increase in SN risk, not a decrease in risk as would be expected from decreased SAMHD1 expression. Further work is required to understand the functional cause of the *SAMHD1* SNP associations with SN observed in this study.

Domingo et al. [41, 42] found associations in Caucasians between low expression *TYMS* genotypes in combination with *MTHFR* polymorphisms, and the development of several d4T-related toxicities, including lipodystrophy, sensory neuropathy and pancreatitis. They hypothesized that normal / high levels of MTHFR would cause decreased levels of thymidylate synthetase (causing lower *de novo* thymidine production) and therefore increased d4T toxicity, while low levels of MTHFR could associate with decreased d4T toxicity [41]. Our results showed a significant association between *MTHFR* SNP rs1801131 and SN, supporting the suggestion of a role of variation in this gene and ART toxicity. SNP rs1801131 or A1298C causes a glu429-to-ala (E429A) substitution which decreases MTHFR activity [43], which theoretically would cause increased thymidylate synthetase levels and decreased d4T toxicity. This is consistent with our finding of a protective effect of rs1801131 against SN.

Ribonucleotide reductase (RNR) catalyses the formation of deoxyribonucleotides from ribonucleotides [44] and therefore plays a critical role in *de novo* DNA synthesis and cell proliferation The tetrameric enzyme is composed of large RNR1 and small RNR2 subunits encoded by the *RRM1* or *RRM2* genes. The RNR2b isoform encoded by the *RRM2B* gene is induced by p53 and controls mtDNA synthesis in non-proliferating cells [45]. We found an association between *RRM2B* SNP rs16918482 and SN in a multivariable model. This SNP is in the 3’ UTR of the gene with no known function. In the 1000 genomes data from the Luhya from Kenya, this SNP tagged 25 other SNPs in *RRM2B* and further work to identify the causal variant underlying association with SN is required. Other mutations in *RRM2B* are known to exist that cause severe mtDNA depletion [45], which is also a characteristic of ART-induced mitochondrial toxicities.

The current study also documents the frequencies of ten ADME variants and 16 variants in the thymidine synthesis pathway in the South African black population and adds to the growing literature on the pharmacogenetic characterization of African populations [46]. To our knowledge, this is the first report on frequencies for 25 of these SNPs in Bantu-speaking Southern Africans. Genetic studies in South Africans are especially important given the high level of genetic diversity and low levels of linkage disequilibrium that exist in South African populations [47], and because, despite shared genetic ancestry, data in existing Hapmap populations cannot be directly extrapolated to represent South African populations.

This study had several weaknesses. The sample size was relatively small. Without statistical correction for multiple testing, it is possible some of these findings are false positives. The SNPs identified in this study had weak effect sizes and may act in combination with other risk alleles for SN previously identified in this cohort [12–14]. The findings in this study may not be easily replicated nor relevant to d4T treatment tailoring since d4T has been phased out of use in South Africa and globally. However the ADME variants described here may play a role in efficacy and toxicity of other antiretroviral drugs that are transported by the same drug transporters. Our data for the thymidine synthesis pathway may contribute to further understanding of pharmacogenetics of other thymidine analogues such as those used in treatment of hepatitis B or cancer, or pharmacogenetics of other treatments such as folate or uridine supplements. In addition, whilst pharmacogenetics is often used to identify individuals at high risk of side effects, it could also offer the potential to identify individuals who can safely use drugs that otherwise are considered toxic, such as d4T.

In conclusion, variation in genes relating to d4T transport and metabolism, as well as genetic variation in the thymidine synthesis pathway may influence occurrence of d4T-related SN. These data contribute to the characterization of African pharmacogenetic variation and its role in adverse response to antiretroviral therapy.

## Supporting information

S1 Table Associations of 26 SNPs with SN in multivariable models, calculated in gPLINK using logistic regression taking into account age and height. (DOCX)
