# Supplementary material for: Pharmacogenetic variation influences sensory neuropathy occurrence in Southern Africans treated with stavudine-containing antiretroviral therapy
Source: PLoS One. 2018 Oct 1;13(10):e0204111. doi: 10.1371/journal.pone.0204111 (PMC6166924; doi:10.1371/journal.pone.0204111)
Supplement: S1 Table — (DOCX) [file pone.0204111.s001.docx]

**Supplementary data.**

S1 Table. Associations of 26 SNPs with SN in multivariable models, calculated in gPLINK using logistic regression taking into account age and height.

Both P and Pemp values are shown, Pemp are from 1000 simulations. Significant Pemp values < 0.05 are highlighted in grey.

| **GENE** | **SNP** | **frequency** | **frequency** | **ALLELIC MODEL** | | | **DOMINANT MODEL** | | | **RECESSIVE MODEL** | | | **GENOTYPIC MODEL** | | |
| --- | --- | --- | --- | --- | --- | --- | --- | --- | --- | --- | --- | --- | --- | --- | --- |
|  |  | **with SN** | **without SN** | **OR** | **P** | **PEMP** | **OR** | **P** | **PEMP** | **OR** | **P** | **PEMP** | **OR** | **P** | **PEMP** |
| *MTHFR* | rs1801131 | 0.127 | 0.165 | 0.644 | 0.129 | 0.149 | 0.681 | 0.218 | 0.249 | 0.122 | 0.146 | **0.002** | 0.334 | 0.131 | **0.001** |
| *MTHFR* | rs1801133 | 0.071 | 0.069 | 0.994 | 0.987 | 0.990 | 0.913 | 0.820 | 0.840 | 1.814 X10^9^ | 0.999 | 0.437 | 42120 | 0.999 | 0.420 |
| *RRM2* | rs7574663 | 0.078 | 0.092 | 0.767 | 0.459 | 0.386 | 0.729 | 0.413 | 0.387 | 1.108 | 0.945 | 0.399 | 1.014 | 0.985 | 0.490 |
| *ABCC5* | rs3749442 | 0.228 | 0.184 | 1.276 | 0.332 | 0.347 | 1.211 | 0.505 | 0.499 | 2.747 | 0.243 | 0.284 | 1.691 | 0.228 | 0.245 |
| *ABCG2* | rs12505410 | 0.142 | 0.098 | 1.546 | 0.170 | 0.218 | 1.637 | 0.147 | 0.167 | 1.244 | 0.864 | 0.735 | 1.178 | 0.798 | 0.717 |
| *ABCG2* | rs2725252 | 0.190 | 0.197 | 1.068 | 0.794 | 0.822 | 1.054 | 0.855 | 0.844 | 1.272 | 0.754 | 0.794 | 1.134 | 0.745 | 0.760 |
| *ABCG2* | rs3114018 | 0.439 | 0.391 | 1.264 | 0.274 | 0.188 | 1.398 | 0.277 | 0.257 | 1.283 | 0.527 | 0.532 | 1.244 | 0.323 | 0.330 |
| *ABCG2* | rs2622604 | 0.141 | 0.120 | 1.316 | 0.344 | 0.347 | 1.294 | 0.436 | 0.449 | 2.321 | 0.388 | 0.326 | 1.559 | 0.364 | 0.305 |
| *DHFR* | rs1650723 | 0.060 | 0.064 | 0.889 | 0.782 | 0.743 | 0.889 | 0.782 | 0.776 | NA | NA | 1.000 | NA | NA | 1.000 |
| *RRM2B* | rs16918482 | 0.060 | 0.069 | 0.980 | 0.958 | 0.931 | 1.011 | 0.980 | 0.970 | 0.653 | 0.772 | **0.001** | 0.810 | 0.774 | **0.002** |
| *SLC28A3* | rs7853758 | 0.508 | 0.491 | 1.105 | 0.613 | 0.693 | 1.216 | 0.543 | 0.546 | 1.072 | 0.829 | 0.796 | 1.105 | 0.614 | 0.627 |
| *SLC28A3* | rs4877847 | 0.463 | 0.477 | 0.863 | 0.463 | 0.465 | 0.651 | 0.181 | 0.166 | 1.070 | 0.843 | 0.848 | 0.879 | 0.526 | 0.530 |
| *RRM1* | rs1465952 | 0.474 | 0.482 | 1.009 | 0.963 | 0.980 | 1.199 | 0.557 | 0.552 | 0.843 | 0.595 | 0.620 | 1.001 | 0.996 | 0.997 |
| *RRM1* | rs11030918 | 0.214 | 0.184 | 1.164 | 0.539 | 0.485 | 1.085 | 0.778 | 0.777 | 2.426 | 0.287 | 0.301 | 1.557 | 0.291 | 0.315 |
| *RRM1* | rs12806698 | 0.022 | 0.019 | 1.473 | 0.580 | 0.535 | 1.473 | 0.580 | 0.549 | NA | NA | 1.000 | NA | NA | 1.000 |
| *RRM1* | rs1042927 | 0.175 | 0.188 | 0.915 | 0.743 | 0.634 | 0.889 | 0.683 | 0.705 | 1.360 | 0.805 | 0.710 | 1.139 | 0.836 | 0.713 |
| *SLC28A1* | rs2290272 | 0.218 | 0.271 | 0.746 | 0.215 | 0.168 | 0.632 | 0.104 | 0.114 | 1.229 | 0.745 | 0.752 | 0.992 | 0.981 | 0.980 |
| *SLC28A1* | rs8187758 | 0.136 | 0.178 | 0.753 | 0.302 | 0.327 | 0.601 | 0.103 | 0.107 | 4.344 | 0.201 | 0.164 | 1.890 | 0.270 | 0.249 |
| *SLC28A1* | rs2242046 | 0.034 | 0.014 | 2.106 | 0.284 | 0.238 | 2.106 | 0.284 | 0.245 | NA | NA | 1.000 | NA | NA | 1.000 |
| *TK2* | rs3743712 | 0.377 | 0.407 | 1.005 | 0.981 | 0.951 | 0.853 | 0.580 | 0.576 | 1.325 | 0.450 | 0.481 | 1.069 | 0.742 | 0.716 |
| *TK2* | rs11859474 | 0.261 | 0.266 | 1.107 | 0.639 | 0.663 | 0.961 | 0.887 | 0.889 | 2.064 | 0.175 | 0.194 | 1.386 | 0.231 | 0.255 |
| *TK2* | rs2288399 | 0.149 | 0.156 | 0.934 | 0.794 | 0.861 | 0.970 | 0.922 | 0.914 | 0.691 | 0.617 | 0.687 | 0.834 | 0.625 | 0.661 |
| *SAMHD1* | rs8124728 | 0.175 | 0.162 | 0.915 | 0.730 | 0.723 | 1.140 | 0.664 | 0.658 | 0.178 | **0.047** | **0.011** | 0.445 | 0.063 | **0.013** |
| *SAMHD1* | rs1291142 | 0.214 | 0.252 | 0.836 | 0.443 | 0.495 | 0.709 | 0.224 | 0.215 | 1.531 | 0.511 | 0.492 | 1.141 | 0.688 | 0.695 |
| *SAMHD1* | rs1891643 | 0.187 | 0.188 | 1.115 | 0.668 | 0.693 | 0.947 | 0.852 | 0.852 | 5.089 | 0.075 | **0.017** | 2.182 | 0.089 | 0.053 |
| *SLC19A1* | rs1051266 | 0.336 | 0.280 | 1.485 | 0.076 | 0.099 | 1.688 | 0.064 | 0.065 | 1.470 | 0.443 | 0.480 | 1.372 | 0.226 | 0.222 |
